# Challenges in Steroid and Anticoagulant Therapy in Severe COVID-19 Pneumonia: A Prospective Study

**DOI:** 10.3390/antibiotics10101214

**Published:** 2021-10-06

**Authors:** Alaa Thabet Hassan, Alaa E. Abd Elmoniem, Marwa Mahmoud Abdelrady, Mona Embarek Mohamed, Mohamed A. Mokhtar, Abdelhalim A. Elsherif, Ghada Mohamed Saied, Soheir M. Kasem

**Affiliations:** 1 Chest Department, Faculty of Medicine, Assiut University, 71515 Assiut, Egypt; alaathabet35@aun.edu.eg; 2 Internal and Critical Care Medicine Department, Faculty of Medicine, Assiut University, 71515 Assiut, Egypt; alaaelmoniem@aun.edu.eg (A.E.A.E.); soheir@aun.edu.eg (S.M.K.); 3 Anesthesia and ICU Department, Faculty of Medicine, Assiut University, 71515 Assiut, Egypt; 4 Medical Microbiology and Immunology Department, Faculty of Medicine, Assiut University, 71515 Assiut, Egypt; monaembarek@aun.edu.eg (M.E.M.); elmokhtarma@aun.edu.eg (M.A.M.); 5 Gastroenterology and Hepatology Department, Faculty of Medicine, Al-Azhar University, 71515 Assiut, Egypt; Haleem1elsherif@gmail.com; 6 Department of Clinical Pathology Department, Faculty of Medicine, Assiut University, 71515 Assiut, Egypt; Ghadasaied11@med.aun.edu.eg

**Keywords:** anticoagulant, corticosteroids, COVID-19, D-dimer, ferritin, RT-PCR

## Abstract

Background: As COVID-19 has neither a standard treatment protocol nor guidelines, there are many treatment protocols for anti-inflammatory corticosteroids and anti-coagulations for severe COVID-19 pneumonia patients. This study aimed to assess the most suitable modality in this high-risk group. Methods: A prospective, experimental study design was adopted that included 123 severe COVID-19 pneumonia patients admitted at Assiut University Hospital. Patients were divided into three groups according to a combined corticosteroid and anticoagulants therapy protocol. Group A included 32 patients, group B included 45 patients, and group C included 46 patients. Assessment of cases was conducted according to the treatment type and duration, weaning duration from oxygen therapy, length of hospital and ICU stay, and complications during treatment. Three months follow-up after discharge was performed. Results: the three patient groups showed significant differences regarding the 3-month outcome, whereas Group C showed the highest cure rate, lowest lung fibrosis, and lowest mortality rate over the other two groups. The in-hospital outcome, the development of pulmonary embolism, bleeding, hematoma, acute kidney disease, and myocardial infarction showed a significant difference between groups (*p* values < 0.05). Mortality predictors among severe COVID-19 patients by multivariable Cox hazard regression included treatment modality, history of comorbid diseases, increased C reactive protein, high neutrophil-lymphocyte ratio, and shorter ICU and hospital stay. Conclusion: the use of combined methylprednisolone and therapeutic Enoxaparin, according to a flexible protocol for COVID-19 patients with severe pneumonia, had two benefits; the prevention of disease complications and improved clinical outcome.

## 1. Introduction

COVID-19 was first isolated in Wuhan, China, in December 2019 [1]. It was declared by the WHO in January 2020 as a global emergency of international concern, and, on 11 March 2020, it was announced as a global pandemic [2]. The virus is called severe acute respiratory syndrome coronavirus-2 (SARS-CoV-2), and the disease it causes has become known as coronavirus disease 2019 (COVID-19) [2]. Symptomatic cases of COVID-19 usually presented with fever, cough, dyspnea, and muscle pain, with a ground-glass appearance in the base of the lungs under radiography, although asymptomatic cases have been also recorded [3]. SARS-CoV-2 is a novel member of the coronavirus family. It is an enveloped virus that shares genetic homology with SARS-related coronaviruses (SARSr-CoV) and Middle East respiratory syndrome-related coronaviruses (MERSr-CoV) [1]. Rapid and highly specific diagnostic tools are critical for case detection and treatment. Viral culture for the detection of acute cases is not practical, as it lasts many days with a requirement for biosafety level three, which is unavailable in the majority of health facilities, and serological tests are not validated and there may be a cross-reaction with other coronaviruses [3]. The diagnosis of COVID-19 is based mainly on medical history, clinical signs and symptoms, and radiological findings, which are then confirmed by the detection of viral nucleic acid by reverse-transcriptase polymerase chain reaction (RT-PCR) for respiratory specimens [4]. No specific antiviral drug has been approved; all the choices of medications come from previous experience in treating SARS, Middle East respiratory syndrome coronavirus (MERS-CoV), or other new influenza viruses. Treatments are based on a respiratory support plus symptom relief. Until recently, no solid evidence had been reported on the efficacy of any specific treatment for suspected or confirmed COVID-19 cases [5]. The Surviving Sepsis Campaign (SSC) guidelines on dealing with adults with severe COVID-19 recommends using corticosteroids in mechanically ventilated patients and those with acute respiratory distress syndrome. Also, in COVID-19 patients with refractory shock, low-dose, short-course corticosteroids are suggested [1]. However, the role of steroids in treating cases of COVID-19 pneumonia is not explicitly addressed in the literature. Moreover, the Infectious Diseases Society of America’s (IDSA) guideline panel advised against the use of corticosteroids that were widely used in China to prevent the development of acute respiratory distress syndrome (ARDS) in patients with COVID-19 pneumonia [6]. As no standard treatment for COVID-19 has been approved, and there are many treatment protocols for anti-inflammatory corticosteroids and anti-coagulants for severe COVID-19 pneumonia cases, this study aims to assess the most suitable modality in this high-risk group.

## 2. Methods

### 2.1. Study Design

This study is prospective and experimental. Patients who had confirmed severe COVID-19 pneumonia with clinical and radiological evidence, admitted at Assiut University Hospital Critical Care (departments of Internal Medicine, Critical Care, Chest). It follows the Helsinki Declaration and was registered at ClinicalTrials.gov (NCT05021588).

### 2.2. Diagnostic Criteria for Severe COVID-19 Pneumonia

Patients were diagnosed as having COVID-19 by both chest imaging, to confirm lung involvement 9X ray and computed tomography [CT] chest)and RT-PCR test [7]. Case inclusion criteria were respiratory distress (respiratory rate [RR] > 30 breaths/min at rest), mean oxygen saturation ≤93%, ratio of arterial oxygen partial pressure to fractional inspired oxygen (PaO_2_/FiO_2_) ≤300 mmHg, and >50% lung involvement, determined by imaging within 24 to 48 h of admission [8].

### 2.3. Patients Were Submitted for

#### 2.3.1. History, Clinical, and Radiological Assessment

Full histories were taken from the patients or their guardian including age, occupation, smoking habits, history of the present illness, previous hospital admission, history of drug intake, history of exposure to suspected patients, traveling history, and history of comorbid diseases. Full clinical examinations were performed including documenting general signs, vital signs and body mass index (BMI), conducting local chest and cardiac examinations, echocardiography (ECG), and local abdominal examinations. Arterial blood gases were measured as recommended by the Clinical and Laboratory Standards Institute (CLSI) [9]. Chest radiology was performed to detect signs of pulmonary infiltration and pneumonia, including CT chest with contrast, and tests needed in clinical situations, like CTs of the brain or abdomen, were conducted accordingly.

#### 2.3.2. Laboratory Tests

Collection of blood samples was conducted to measure complete blood counts (CBC), cardiac enzymes, kidney and liver function tests, erythrocyte sedimentation rate (ESR), C-reactive protein (CRP) levels, and other laboratory metrics according to patients’ clinical situations. Ferritin levels were measured by commercially available ELISA kits (MyBioSource, USA), and a rapid sensitive D-dimer test was used for measuring D-dimer levels. Microbiological cultures of sputum and blood samples were conducted. The detection of the viral genome was performed by real-time RT-PCR diagnostic panel, as recommended by the Center for Disease Control and Prevention (CDC) using an Applied Biosystems (ABI) 7500 Real-time PCR instrument.

#### 2.3.3. Management Policy for Severe COVID-19 Pneumonia Patients and Prospective Design

All cases with severe COVID-19 pneumonia received the available antiviral drugs, empirically validated antibiotics, and the standard co-morbidities treatments. Treatment type and duration, time needed for oxygen therapy weaning, hospital and intensive care unit (ICU) stays, and complications or adverse events during treatment including bleeding (either orifical or gastrointestinal [GIT]), hematoma formation, development of pulmonary embolism, myocardial infarction, secondary bacterial infections, and other complications were assessed.

After fulfilling the inclusion criteria, patients were randomly allocated into one of three groups using a computer-generated randomization table carried on 123 eligible patients:

- Group A: Patients with severe COVID-19 pneumonia, treated with dexamethasone and anticoagulants according to the D-dimer levels.
- Group B: Patients with severe COVID-19 pneumonia, treated with prednisolone or methylprednisolone and anticoagulants according to D-dimer levels.
- Group C: Patients with severe COVID-19 pneumonia, treated with prednisolone or methylprednisolone and anticoagulants according to the flexible protocol.

##### Treatment Policies

Corticosteroids: 

− Dexamethasone: a dosage of 6 mg was administered in a once-daily oral (liquid or tablet) or intravenous (IV) preparation, to be used for up to 10 days, until discharged, or until the patient became asymptomatic.
− Prednisolone or equivalent methylprednisolone: prednisolone, at dosage of 60 mg, was administered to patients on non-rebreathing masks equal to or less than 10 L, intravenously or orally, divided into three equal doses per day For patients on non-rebreathing masks equal to or greater than 10 L or mechanical ventilation, the dose was 120 mg infusion over 24 h. After clinical and radiological improvement and the reduction of FiO_2_, the dose was titrated down by 25%, and then by 50%, according to clinical response and oxygen requirement. The dose was decreased to 40 mg orally when O_2_ requirements fell to 6 L or less. When patients were ready for discharge and O_2_ saturation was more than 93% on room air, the dose was 30 mg OD in the morning, gradually decreased by 5 mg every 7 days. Some patients in the first group were discharged on oxygen therapy.

Anticoagulation: 

− Anticoagulation According to D-dimer Level

- if D-dimer was less than 1 µg/mL
- if bodyweight was less than 100 kg, Enoxaparin at 40 mg was given subcutaneously (SC) daily.
- if bodyweight was between100 kg and 150 kg, SC Enoxaparin at 40 mg was given twice daily.
- if bodyweight was more than 150 kg, SC Enoxaparin at 60 mg was given twice daily.
- if D-dimer more than 1 µg/mL
- if bodyweight was less than 100 kg, SC Enoxaparin at 40 mg was given twice daily.
- if bodyweight was between 100 kg and 150 kg, SC Enoxaparin at 80 mg was given twice daily.
- if bodyweight was more than 150 kg, SC Enoxaparin at 120 mg was given twice daily.

− Anticoagulation According to the Flexible Protocol

All patients with severe COVID-19 pneumonia received at least Enoxaparin 1 mg /KG twice daily, up to 80 mg twice daily; to patients with thrombocytopenia, Fondaparinux was administered at a dose of 5 mg SC for patients less than 50 KG and 7.5 mg for patients more than 50 KG BW. After clinical and radiological improvement and reduction of FiO_2_, the dose was titrated down by 25%, and then by 50%. The dose of Enoxaparin was decreased to 40 mg SC, once daily, and the dose of Fondaparinux was decreased to 25 mg once daily when O_2_ requirements become 6 L or less; finally, on discharge, patients were discharged on rivaroxaban 10 mg, or any other NOACS, once daily for 1 month.

#### 2.3.4. Follow-Up for Surviving Patients for 3 Months after Discharge from the Hospital

The outcome definitions were as follows.

1. Cure: considered if patients returned to their usual activities before COVID-19 with O_2_ saturations 96% or above, for patients with no history of respiratory failure before COVID-19, or for patients who returned to their baseline saturations with clearance of the chest by X-ray or chest CT.
2. Death.
3. Mild residual fibrosis with desaturation from 90 to 95 with residual lung fibrosis, including mid-reticular infiltrates but not requiring long-term O_2_ therapy.
4. Moderate lung infiltrations with desaturation from 85 to 89 requiring O_2_ therapy and further follow-up, with bilateral residual lung infiltrates but occupy less than 50% of both lung fields.
5. Severe disability and O_2_ saturation below 84% with the need for long-term oxygen therapy and chest X-ray or CT showing bilateral extensive lung fibrosis of more than 50% of the lung field, with the need for long-term rehabilitation.

### 2.4. Statistical Analysis

SPSS-24 was used for data processing and analysis. Data were expressed in means, standard deviations, medians, inter-quartile range (IQR), and percentages. The Chi-square test was calculated to compare frequencies distribution differences between groups. One-way ANOVA tests were used for comparison of differences in means between groups, with post-hoc tests using Bonferroni corrections for pair wise comparisons. The Kaplan–Meier curve was used to estimate the median survival time. A log-rank test was applied to examine the effect of the explanatory variables on overall survival. Multivariable Cox hazard regression analyses were calculated to investigate the significant factors influencing OS and DFS (hazard ratio -HR-, 95% confidence interval -95% CI-). A *p*-value < 0.05 was considered significant.

### 2.5. Ethical Considerations

The study’s approval was obtained from the Institutional review board of the Assiut University Faculty of Medicine. All participants were asked to sign written consent before enrollment. The consent included an information sheet about the purpose of the study, its benefits, and its drawbacks. It also stated that they are free to participate/withdraw without any obligation. Further, confidentiality was assured. The study was in line with the Declaration of Helsinki.

## 3. Results

A total of 123 severe COVID-19 pneumonia patients were recruited in this study and divided into three groups; group A included 32 patients, group B included 45 patients, and group C included 46 patients.

### 3.1. Baseline Characteristics of the Studied Cohort

Most of our patients were male (73%), mean age was 50.62 ± 16.4; metabolic syndrome was the most prevalent comorbidity in our studied patients, were presented as hypertension (30%); diabetes mellitus (DM) (27.6%); dyslipidemia (22%); and obesity (mean BMI was 28.29 ± 4.8) respectively. Patients with ischemic heart diseases (IHD) were a vulnerable group in our cohort, followed by those with pulmonary diseases, chronic kidney disease (CKD), liver cirrhosis, and cancer (Table 1).

### 3.2. CBC Finding of the Studied Cohort

Our studied patients show lymphopenia and increased NLR; otherwise other CBC parameters were within normal range (Table 2).

### 3.3. Prevalence of Symptoms among Severe COVID-19 Pneumonia Patients

**Figure 1:**
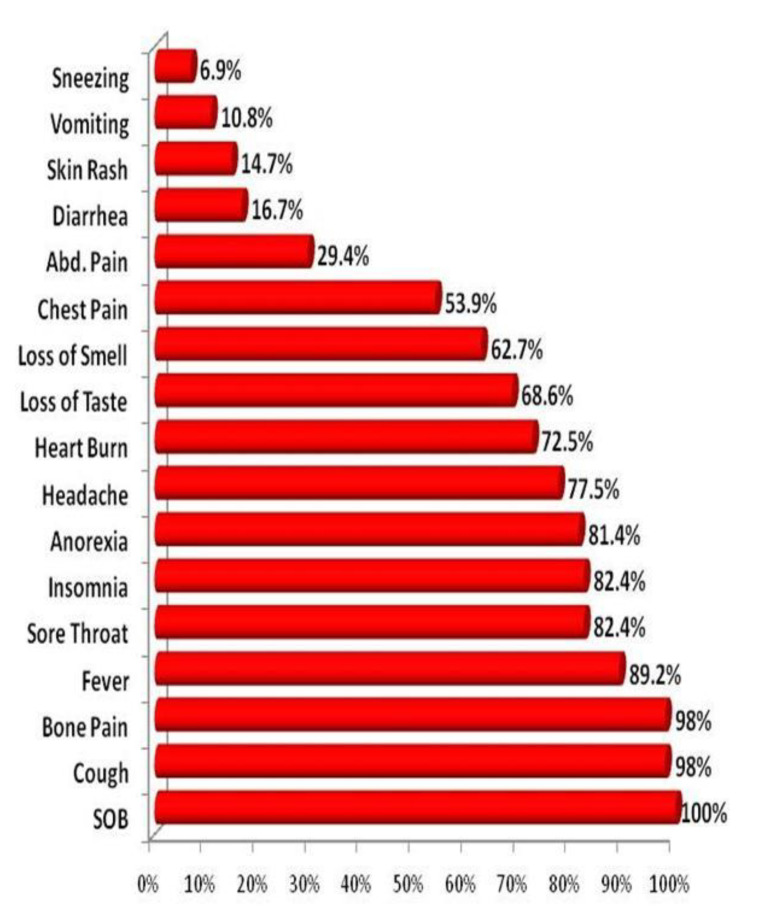
Prevalence of symptoms among COVID-19 severe pneumonia patients. SOB= shortness of breath.

All of our patients presented with shortness of breath (100%); cough and bone pain were the second-most frequent symptom (98%); and sneezing and vomiting were the least recorded symptoms in our severe COVID-19 pneumonia patients (10.8% and 6.9%, respectively) (Figure 1).

### 3.4. Laboratory Panel of the Studied Cohort

Our patients also had elevated serum levels of inflammatory markers. i.e., D-dimer, ferritin, and ESR (Table 3).

### 3.5. Relationship between Disease Outcome/Complication and Treatment Modality

As regards in-hospital outcome, the three groups were comparable in terms of temporary DM, neuromuscular weakness, and secondary bacterial infections (*p* values were 0.457, 0.255, and 0.349, respectively), but there were significant differences between groups as regard pulmonary embolism; six (18.8%) patients in group A developed pulmonary embolism (three, after discharge from hospital and being indicated for readmission and anticoagulation for 6 months) vs eight (17.8%) (Four, after discharge from hospital and being indicated for readmission and anticoagulation for 6 months) patients in group B, while only one (2.2%) patient in group C developed pulmonary embolism (during hospital admission; but it was only segmental and the patient received oral anticoagulant for 3 months) (*p* = 0.02). Bleeding was reported more in group A, where in four (12.5%) patients suffered from bleeding vs five (11.1%) patients in group B, and one patient (2.2%) in group C(*p* = 0.047). Hematoma was also highly recorded in group A; five (15.6%) patients vs six (13.3%) patients in group B, and three (6.5%) patients in group C(*p* = 0.025). Acute kidney injury in Group A was detected in four (12.5%) patients, while five (11.1%) patients in group B and two patients (4.3%) in group C (*p* = 0.031); in all groups, any patient who suffered from bleeding or significant hematoma; anticoagulation cessation were done and resumed after control by 50% of the previous dose. Myocardial infarction was significantly higher in Group A (three patients; 9.4%) vs three (6.7%) patients in group B, while no cases were recorded in group C (*p* = 0.016) (Table 4).

**Figure 2:**
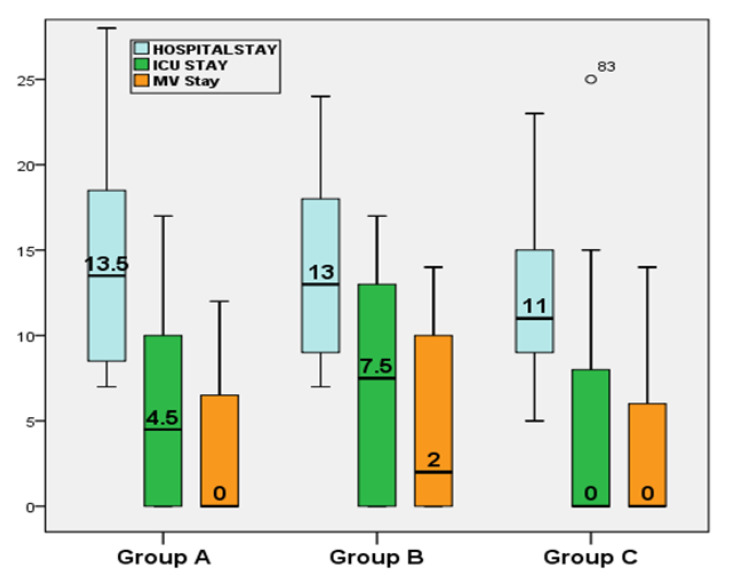
The median hospital stay duration among the COVID-19 treatment groups. ICU = intensive care unit; MV = mechanical ventilation.

The 3-month outcomes (complete cure, lung fibrosis, and death) showed significant difference among groups (*p* = 0.028); Group C showed the highest cure rate, lowest lung fibrosis, and lowest mortality rate; 12 patients (37.5%) vs. 26 (57.8%) and 31 (67.4%) patients in groups A, B, and C, respectively, experienced complete cure; while 14 patients (43.8%) vs. 11 (24.4%) vs. 10 (21.7%) in group A, B, and C, respectively, had lung fibrosis (in group A, six patients had moderate lung fibrosis and oxygen therapy at home for 3 months, eight patients had mild fibrotic bands, and none had sever lung fibrosis; in group B, one patient had moderate lung fibrosis and oxygen therapy at home for 3 months, 10 patients had mild fibrotic bands, and none had sever lung fibrosis; and, in group C, no patients had moderate or severe lung fibrosis or oxygen therapy at home and 10 patients had mild fibrotic bands); six (18.8%) patients died in group A (four due to secondary bacterial infections and sepsis, one due to PE, and one due to MI and cardiac arrhythmias), seven (15.6%) patients died in group B (five due to secondary bacterial infections and sepsis, one due to PE, and one due to MI and cardiac arrhythmias), and five (10.9%) patients died in group C (due to secondary bacterial infections and sepsis) (Table 4 and Figure 2).

### 3.6. Mortality Predictors among Severe COVID-19 Patients

**Figure 3:**
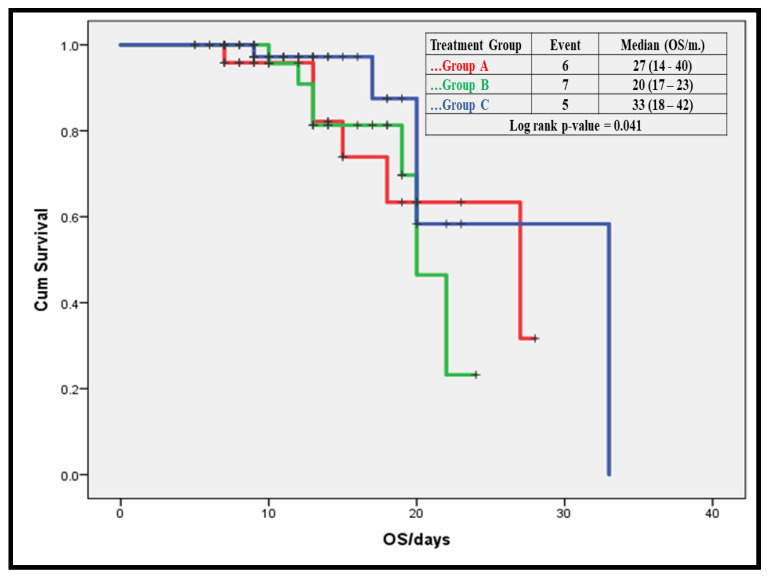
Effect of treatment on overall survival (OS) among the studied severe COVID-19 patients. Treatment modality had a significant effect on OSof our patients; group C showed the longest OS (33 days).

Predictors among our Severe COVID-19 patients by multivariable Cox hazard regression were treatment modality (lowest mortality and long overall survival [OS])in group C; history of comorbid diseases (DM, MI, CKD, COPD, asthma, and lung fibrosis); increased CRP and neutrophil-lymphocyte ratio (NLR); and shorter hospital, ICU, and MV stay (Table 5 and Figure 3).

## 4. Discussion

Patients with COVID-19 pass through three stages: viremia, acute disease(pneumonia phase), and the severe or recovery stage [10]. Those with an intact immune system and without risk factors (old age, comorbidities, etc.) may experience effective immune responses that overcome the virus during the first two stages without immune over-reaction. On the other hand, patients with an immune deficiency may experience greater risk and enter the third stage of severe or critical illness; one with higher mortality [11].

In this study, age, as a factor, did not reach a statistical significance. Indeed, recent data has showed that mortality is not influenced by age itself, but rather by the fitness of patients [12].

COVID-19 management should be based on disease stage, where the best cure opportunity lies between the first and the second phases when clinical progress is observed with evidence of abrupt inflammation and hypercoagulable states. Given that there are no approved antivirals, proper disease-stage timing andconsideration of methods for stopping or slowing disease progression are criticalfor early intervention. For patients in the third stage, there is no definite treatment protocol, other than comprehensive management [13]

The use of anti-inflammatories as adjuvants to antiviral drugs has shown better results, as compared with using either modality alone. This owes to the dual actions of inhibiting SARS-CoV-2 replication and blocking SARS-Co-2 infection-induced pro-inflammatory cytokine production, in vitro [13].

### Clinical Therapeutic Staging Proposal

A protocol for management was proposed based on disease staging, as follows:

- Stage I: Mild (Early Infection): steroid administration during this stage could result in boosting viral replication and perhaps hindering the initiation of immune response.
- Stage II-a: Moderate (Pulmonary Involvement without Hypoxia) and Stage II-b: Moderate (Pulmonary Involvement with Hypoxia): the use of low-dose steroids during the pulmonary stage might be beneficial (by alleviating inflammation severity and thereby preventing a severe hyper-inflammation phase).
- Stage III: Severe (Systemic Hyperinflammation): For patients with a marked hyper-inflammation phase (cytokine storm), higher doses of steroid or targeted immunosuppressive drugs (e.g., tocilizumab) may be necessary to treat the established hyper-inflammation. [14,15]

Dyspnea, in severe cases, develops between five and eight days; in ARDS cases,8–12 days, and, in ICU admission cases, 10–12 days. Rapid deterioration was reported one week after illness onset. Mortality rates in ICU admissions were high (39–72%). Additionally, the median hospital stay among survivors ranged between one and two weeks. ARDS was identified as the major cause of mortality in COVID-19 pneumonia [16,17,18].

For the proper management of cases, a better understanding of the disease pathway is fundamental. Two major immune response processes preceding the third stage of the disease were identified: the first is the macrophage activation syndrome-like state, and the second is the IL-6-driven defective antigen presentation [8].

The unique clinical presentation of CAP, caused by SARS-CoV-2, including abrupt deterioration one week after the first manifestation, supporting the proposal that this disease is driven by a peculiar immune dysfunction, different from that of the inflammatory response. Lymphopenia patterns, with liver malfunction and D-dimer increase, in severe cases, further support this hypothesis [8,19].

It was postulated that IL-6 and IL-8 may cause a hypercoagulable state, leading to scattered fibrin clots, shortening the clot dissolution time, and maximizing the dissolution rate [20]. It was also observed that patients with severe COVID-19 had higher levels of IL-6 [21]. This is supported by the fact that hypercoagulation status in patients may be associated with higher levels of cytokines. This is further supported by pathological observations in critically ill patients, in which a higher frequency of pulmonary micro-thrombosis has been noted [22].

According to the recommendation of the American Society of Hematology (ASH), low-molecular-weight heparin (LMWH) should be considered in *all* COVID-19 cases (including non-critically ill) who require hospital admission, provided it is not contraindicated (active bleeding, platelet count <25 × 10^9^/L). Also, strict monitoring is recommended in severe renal impairment and abnormal PT [23]. Despite the scarcity of the published evidence-based protocols, intermediate-intensity protocols were adopted by many institutions (i.e., use of the usual LMWH dose twice daily) or even a therapeutic-intensity dose strategy for thromboprophylaxis based on local experience [24]. It was found that LMWH exerts an anti-inflammatory response (via IL-6 reduction and lymphocyte% increase) as well as improving the coagulation dysfunction [25].

Why shouldn’t corticosteroids be the 1stline therapy (as these will markedly suppress IL-6 and a raft of other cytokines)?, if a MAS-like state exists and excessive IL-6 levels are detrimental. Although the findings ofWu and colleagues showed a benefit for corticosteroids [24], the consensus is that these should not be used based on clinical experience in MERS-CoV, SARS-CoV, and all other infections, including respiratory syncytial virus and influenza infection, where, collectively, there is evidence for delayed viral clearance [26]. In a multi-center pre/post study examining the effect of using early steroids within a health system, in Michigan, USA (including five hospitals), it was concluded that early implementation of short course methylprednisolone in moderate to severe COVID-19 cases reduced the elevation of the level of care and improved overall clinical result [27].

The current study has shown that methylprednisolone and enoxaparin, in patients admitted with severe COVID-19 pneumonia and according to the flexible protocol (group-C), showed significant favourable outcome, especially in-hospital, for lower thromboembolic complications (i.e., pulmonary embolism and myocardial infarction), lower bleeding tendency (hematoma or orifical, GIT), and AKI; although, there was an insignificant difference between groups with respect to temporary DM and neuromuscular weakness and secondary bacterial infections, and this encouraged the use of methylprednisolone instead of dexamethasone in those critically ill patients with good control of bacterial infection and electrolyte follow up. Enoxaparin, in therapeutic doses, was used in this group as an anti-inflammatory without major or minor bleeding, irrespective of D-dimer level and, obviously, with better outcome.

There was a statistically significant difference between the three groups as regards 3-monthsoutcome in terms of complete cure, lung fibrosis, or death; i.e., Group C showed the highest cure rate, lowest rate of lung fibrosis, and lowest mortality rate. The independent predictors of mortality among the studied cohort of severe COVID-19 cases were: treatment modality (lowest mortality and long overall survival [OS] in group C); history of comorbid diseases (DM, MI, CKD, COPD, asthma, and lung fibrosis), increased CRP and NLR; and longer hospital, IC, and MV stay.

## 5. Study Strengths and Limitations

This study had several strengths, i.e., using a double-blinded RCT design and examining the main predictors for mortality and outcome of the different treatment modalities. However, several limitations were recorded, i.e., this study was a single-center study, which limits its generalizability and representation, as both were limited to our center. Also, MDR, which is an important determinant of mortality especially in severe COVID-19 cases, was not addressed in this study.

## 6. Conclusions and Summary

In conclusion, the early use of corticosteroids in combination with Enoxaparin an inexpensive and readily available anti-inflammatory agent in patients with severe COVID-19 pneumonia cases could prevent adverse disease effects and improve overall outcome. More studies—especially RCTs—are needed to determine the benefit/effectiveness of the use of combined anti-inflammatory (corticosteroids and Enoxaparin) treatment. Trials of this combined modality on COVID-19 patients at different disease stages are also needed. 

This study had shown that the use of combined methylprednisolone and therapeutic Enoxaparin as anti-inflammatory agents, according to a flexible protocol for COVID-19 patients with severe pneumonia, had two benefits; first, is preventing disease complications and hence mortality, and second is improving clinical outcome, both in the short and long terms.

## Figures and Tables

**Table 1:** Baseline characteristics of the studied cohort.

Parameter	Category	(*n* = 123)
Age (years)	mean ± SD	50.62 ± 16.4
	median (IQR)	51.5 (22)
Sex	male	90 (73.2%)
	female	33 (26.8%)
BMI	mean ± SD	28.29 ± 4.8
	median (IQR)	28 (5)
Chronic Disease History:		Number (%)
HTN		37 (30.0%)
DM		34 (27.6%)
Dyslipidaemia		27 (22.0%)
IHD		20 (16.3%)
Asthma		19 (15.4%)
COPD		16 (13.0%)
Cancer		14 (11.4%)
Lung Fibrosis		9 (7.3%)
CKD		6 (4.9%)
Liver Cirrhosis		2 (1.6%)

BMI = body mass index; CKD = chronic kidney disease; COPD = chronic obstructive pulmonary disease; DM = diabetes; HTN = hypertension; IHD = ischemic heart disease; IQR = interquartile range.

**Table 2:** CBC Findings of the studied Cohort.

Parameter	Category	(*n* = 123)
CBC Parameters:		
HGB level (g/dL)	mean ± SD	12.63 ± 1.9
	median (IQR)	12.5 (3.3)
WBCs (*10^3^)	mean ± SD	5.98 ± 2.8
	median (IQR)	5.5 (4)
Platelet (*10^3^)	mean ± SD	246.27 ± 93.2
	median (IQR)	218 (126)
Lymphocytes (%)	mean ± SD	25.32 ± 16.5
	median (IQR)	19 (16)
Neutrophils (%)	mean ± SD	64.70 ± 18.7
	median (IQR)	67 (30)
Monocytes (%)	mean ± SD	9.11 ± 4.2
	median (IQR)	9 (6)
NLR	mean ± SD	4.59 ± 4.0
	median (IQR)	3.5 (3)

CBC = complete blood count; HGB = hemoglobin; IQR = interquartile range; NLR = neutrophil lymphocyte ratio; PLT = platelets; RBC = red blood cells; WBC = white blood cells.

**Table 3:** Other laboratory findings of the studied Cohort.

Parameter	Category	(*n* = 123)
ESR (mm/1sthr)	mean ± SD	19.40 ± 13.8
	median (IQR)	14 (11)
Serum creatinine (mg/dL)	mean ± SD	90.44 ± 15.9
	median (IQR)	91 (21)
Liver function parameters:		
ALT (U/L)	mean ± SD	34.22 ± 18.2
	median (IQR)	28.5 (18)
AST (U/L)	mean ± SD	34.54 ± 14.3
	median (IQR)	31 (21)
D-dimer (μg/mL)	mean ± SD	1.43 ± 0.8
	median (IQR)	1.2 (1)
CRP	mean ± SD	13.94 ± 5.2
	median (IQR)	12 (7)
Ferritin (ng/mL)	mean ± SD	459.55 ± 206.1
	median (IQR)	421 (293)

ALT = alanine transferase; AST = aspartate transferase; CRP = C reactive protein; ESR = erythrocyte sedimentation rate; IQR = interquarantile range.

**Table 4:** Relationship between disease outcome/complication and treatment modality.

	Group A (*n* = 32)	Group B (*n* = 45)	Group C (*n* = 46)	*p*
***Baseline Characteristics:***				
Age (years)	50.71 ± 15.6	46.44 ± 16.6	53.48 ± 16.4	0.177 **
*p* ***	0.334	0.063	0.501	
*Sex (Male/Female)*	24/8	33/11	34/12	0.963
BMI	29.42 ± 4.6	27.14 ± 4.3	28.49 ± 4.9	0.198 **
*p* ***	0.079	0.221	0.441	
***In-hospital outcome:***				
Pulmonary embolism	6 (18.8%)	8 (17.8%)	1 (2.2%)	**0.020**
Bleeding	4 (12.5%)	5 (11.1%)	1 (2.2%)	**0.047**
Hematoma	5 (15.6%)	6 (13.3%)	3 (6.5%)	**0.025**
Secondarybacterial infection	13 (40.6%)	19 (42.2%)	21 (45.7%)	0.349
AKI	4 (12.5%)	5 (11.1%)	2 (4.3%)	**0.031**
MI	3 (9.4%)	3 (6.7%)	0 (0%)	**0.016**
DM	6 (18.8%)	11 (24.4%)	9 (19.6%)	0.457
Neuromuscular weakness	9 (28.1%)	14 (31.1%)	11 (23.9%)	0.255
Hospital stay/days	14.13 ± 1.3	13.63 ± 0.9	12.46 ± 0.8	0.432 **
*p* ***	0.738	0.361	0.234	
ICU stay (days)	5.88 ± 1.2	7.03 ± 1.1	4.52 ± 0.9	0.178 **
*p* ***	0.473	0.070	0.368	
MV duration (days)	3.42 ± 0.9	4.53 ± 0.9	2.96 ± 0.6	0.334 **
*p* ***	0.375	0.142	0.694	
***Three-month outcome after discharge:***				
Cured	12 (37.5%)	26 (57.8%)	31 (67.4%)	0.028 *
Lung Fibrosis	14 (43.8%)	11 (24.4%)	10 (21.7%)	
Dead	6 (18.8%)	7 (15.6%)	5 (10.9%)	

AKI = acute kidney injury; DM = diabetes mellitus; ICU = intensive care unit; MI = myocardial infarction; MV = mechanical ventilation. * Chi-square test was used to compare the proportion difference between groups, ** ANOVA test was used to compare means among groups, and *** post-hoc test with Bonferroni correction was used for pairwise comparisons. The bolds show statistically significant values.

**Table 5:** Mortality predictors among severe COVID-19 pneumonia patients; multivariable Cox hazard regression for overall survival (OS).

Variable	HR	95% CI	*P*
Age (years)	1.042	0.993–1.093	0.093
Sex (male)	2.099	0.989–1.111	0.303
Treatment Modality:			
Group A			**0.042**
Group B	0.917	0.701–1.867	0.204
Group C	0.716	0.555–0.923	**0.010**
History of Asthma	4.516	1.849–11.035	**0.031**
History of COPD	14.678	1.610–33.785	**0.017**
History of DM	2.296	1.392–3.788	**0.001**
History of CKD	25.202	5.811–59.863	**0.003**
History of Lung Fibrosis	12.159	1.517–37.467	**0.019**
History of MI	6.878	1.923–51.271	**0.022**
CRP	1.181	1.012–1.377	**0.034**
NLR	1.062	1.008–1.348	**0.046**
Hospital stay/days	0.555	0.432–0.715	**≤0.001**
ICU stay/days	0.753	0.617–0.919	**0.005**
MV duration/days	0.790	0.650–0.960	**0.018**

CI = confidence interval; CKD = chronic kidney disease; COPD = chronic obstructive pulmonary disease; CRP = C reactive protein; DM = diabetes mellitus; HR = hazard ratio; ICU = intensive care unit; MI = myocardial infarction; MV = mechanical ventilation; and NLR = neutrophil lymphocyte ratio. The bolds show statistically significant values.

## Data Availability

The data that support the findings of this study are available on request from the corresponding author. The data are not publicly available due to privacy or ethical restrictions.
